# Difficulty in diagnosing intracranial infection caused by *Mycobacterium avium* in an AIDS patient: case report and review of the literature

**DOI:** 10.1186/s13000-024-01515-z

**Published:** 2024-07-09

**Authors:** Mengyan Wang, Yahui Cui, Jinchuan Shi, Jun Yan

**Affiliations:** 1 Department II of Infectious Diseases, Xixi Hospital of Hangzhou, Hangzhou Xixi Hospital Affiliated to Zhejiang University of Traditional Chinese Medicine, Hangzhou, China; 2 Department of Neurosurgery, Xixi Hospital of Hangzhou, Hangzhou Xixi Hospital Affiliated to Zhejiang University of Traditional Chinese Medicine, Hangzhou, China

**Keywords:** Mycobacterium avium, MAC, CNS, HIV, Case report

## Abstract

**Background:**

Mycobacterium avium complex (MAC) is an uncommon clinical pathogen, especially in the central nervous system (CNS), and carries a poor prognosis. MAC infections commonly present as immune reconstitution disease (IRD) in HIV patients. Herein, we report a case of intracranial infection caused by MAC in an AIDS patient without disseminated MAC (DMAC) and immune reconstitution inflammatory syndrome (IRIS).

**Case presentation:**

A 31-year-old HIV-positive male presented us with progressively worsening CNS symptoms, and neuroimaging revealed ring-enhancing lesions. The intracranial lesions worsened after the empirical therapy for toxoplasma encephalitis and fungal infection. Due to the rapid progression of the disease, the patient died. Mycobacterium avium was the only pathogen in brain tissue after cultures and molecular biology tests.

**Conclusion:**

MAC infection in CNS is challenging to diagnose in HIV patients. Our findings emphasize that obtaining tissue samples and applying molecular biology methods is essential to help diagnose the patient as soon as possible to receive adequate treatment.

## Introduction

Mycobacterium avium complex (MAC) includes *M. avium, M. intracellulare, M. arosiense, M. bouchedurhonense, M. chimaera, M. colombiense, M. marseillense, M. timonense, M. vulneris, and M. yongonense*. Among them, *M. avium, M. intracellulare, and M. chimaera* are the most important human pathogens, and they can find from water, house dust, and soil [1]. Mycobacterium avium complex (MAC) was the most common etiology, followed by M. fortuitum and M. abscesses (34.8%, 21.4%, and 15.2%, respectively). The overall case fatality rate was 37.5% [2]. Since the prevalence of acquired immunodeficiency syndrome (AIDS), NTM infection has increased [3]. Furthermore, primarily, central nervous system (CNS) infections involving MAC are seen as opportunistic infections in AIDS patients with a severely depressed CD4 count (< 50 cells/µl) [4]. However, intracranial infection involving MAC in AIDS patients is rare without disseminated MAC (DMAC) and immune reconstitution inflammatory syndrome (IRIS), and timely diagnosis is difficult.

Here, we describe a case of intracranial infection caused by *Mycobacterium avium* in an AIDS patient. We also review clinical features, diagnostic methods, and outcomes of other cases caused by MAC described in the literature.

## Case report

The patient was a 31-year-old male who confirmed the HIV infection three weeks ago and did not start the combined antiretroviral therapy (cART) admitted to the hospital on September 20, 2022. He felt throat pain, occasionally dysphagia, accompanied by double vision, and denied fever, nasal congestion, and night sweats five days ago. No history of trauma and neurosurgery. Physical examination: white patches can be seen in the oral mucosa, the rectus muscle of the left eye was partially paralyzed, and muscle strength was expected in the extremities. Blood count and blood biochemistry were normal. Cryptococcal capsular antigen, 1-3-β-D glucan detection, aspergillus galactomannan detection, and Mycobacterium tuberculosis interferon-gamma release assays were negative in blood. The viral load of HIV-RNA was 2.80 × 10^5 copies/mL, and the CD4 + T-cell count was 5 cells/µl. The patient was presumed to have fungal esophagitis and then treated with a fluconazole needle 0.4 g QD.

On September 28, 2022, the patient gradually developed drooping eyes, obviously of the right eye, dizziness, and diplopia. Physical examination: spontaneous rotating nystagmus, diplopia (up, down, left, right), positive knee shin test. Subsequent brain magnetic resonance imaging (MRI) showed multiple abnormal signal foci on both sides of the para ventricle, left cerebellar hemisphere, and left pontine arm. On September 29, his cerebrospinal fluid pressure was normal. Cerebrospinal fluid (CSF) had nucleated cell count 2*10^6/L; glucose 2.9mmol/L, protein 1558 mg/L, chlorine 118mmol/L, ADA 1.4U/L, lactate dehydrogenase 21U/L. Cryptococcus neoformans smear, Cryptococcal antigen, Mycobacterium tuberculosis X-pert, EBV antigen, and syphilis RPR were all negative. No acid-fast bacteria were detected under the smear microscope, and there was no finding in fluorescent staining. The bacterial and fungal cultures were negative. Toxoplasma gondii IgM was 0.04 AU/ml, and toxoplasma gondii IgG < 0.600 IU/mL. The patient was presumed toxoplasmosis encephalopathy, treated with clindamycin 0.6 g Q8H and azithromycin 0.5 g QD, then azithromycin monotherapy 0.5 g QD. The results of mNGS in CSF revealed EBV (4669 series), fine cyclic virus (872 series), and CMV (32 series). Because viral encephalitis cannot be excluded, a ganciclovir needle 0.25 g Q12H was used.

Since October 8, the patient had a fever without obvious chills. Routine blood cultures and acid-fast bacilli blood cultures were negative. WBC count was 4.05*10^9/L; neutrophil ratio was 84.0%; rapid C-reactive protein was 76.44 mg/L, and blood amyloid A > 550.00 mg/L. We considered a bacterial infection of the pharynx in the patient. Piperacillin-tazobactam needle 4.5 g intravenously for Q8h was used. Subsequent throat tissue cultures were Streptococcus constellation and actinomyces caries, and antibiotics were adjusted to ceftriaxone 2 g intravenously QD according to drug sensitivity.

**Fig. 1:**
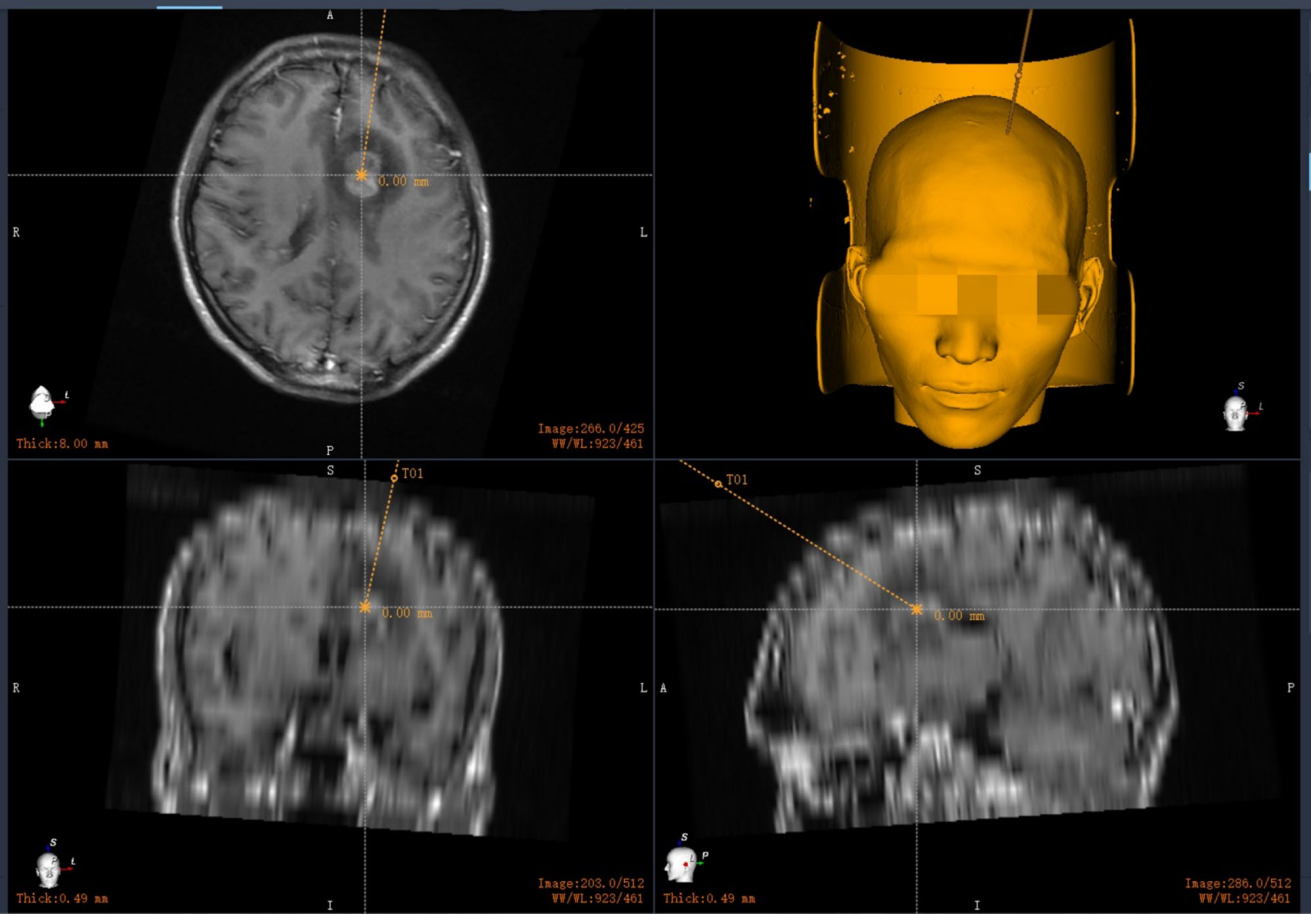
In the figure, the crosshair anchor is the target of the biopsy tissue site, and the yellow dotted line is the puncture path

On October 10, an MRI of the brain progressed significantly. Two days later, a stereotactic robotic brain biopsy was performed under general anesthesia (Fig. 1). Coagulated necrotic tissue (left frontal lobe) had a small amount of lymphocyte infiltration, which is considered an EBV infection with an atypical proliferation of small lymphocytes, and lymphoma needs to be excluded. Immunohistochemistry/special staining: CD10(-) CD20(B cells+) CD3(T cells+) EBV (+) Ki-67(-) MUM1(-) PAX5 (scattered +) PAS (-) Acid-fast (-) Silver hexaammonia staining (-). Mycobacterium tuberculosis X-pert and bacterial and fungal cultures of brain tissue were negative. PMseq test of high-throughput gene detection of brain tissue had EBV (6949 sequences) and fine cyclic virus (191 sequences). Based on these results, we started cART (Biktarvy 1 capsule once daily combined with Albuvirtide injection 0.32 g intravenous drip once daily for three days, followed by one week once adjuvant antiviral therapy) on October 20.

**Fig. 2:**
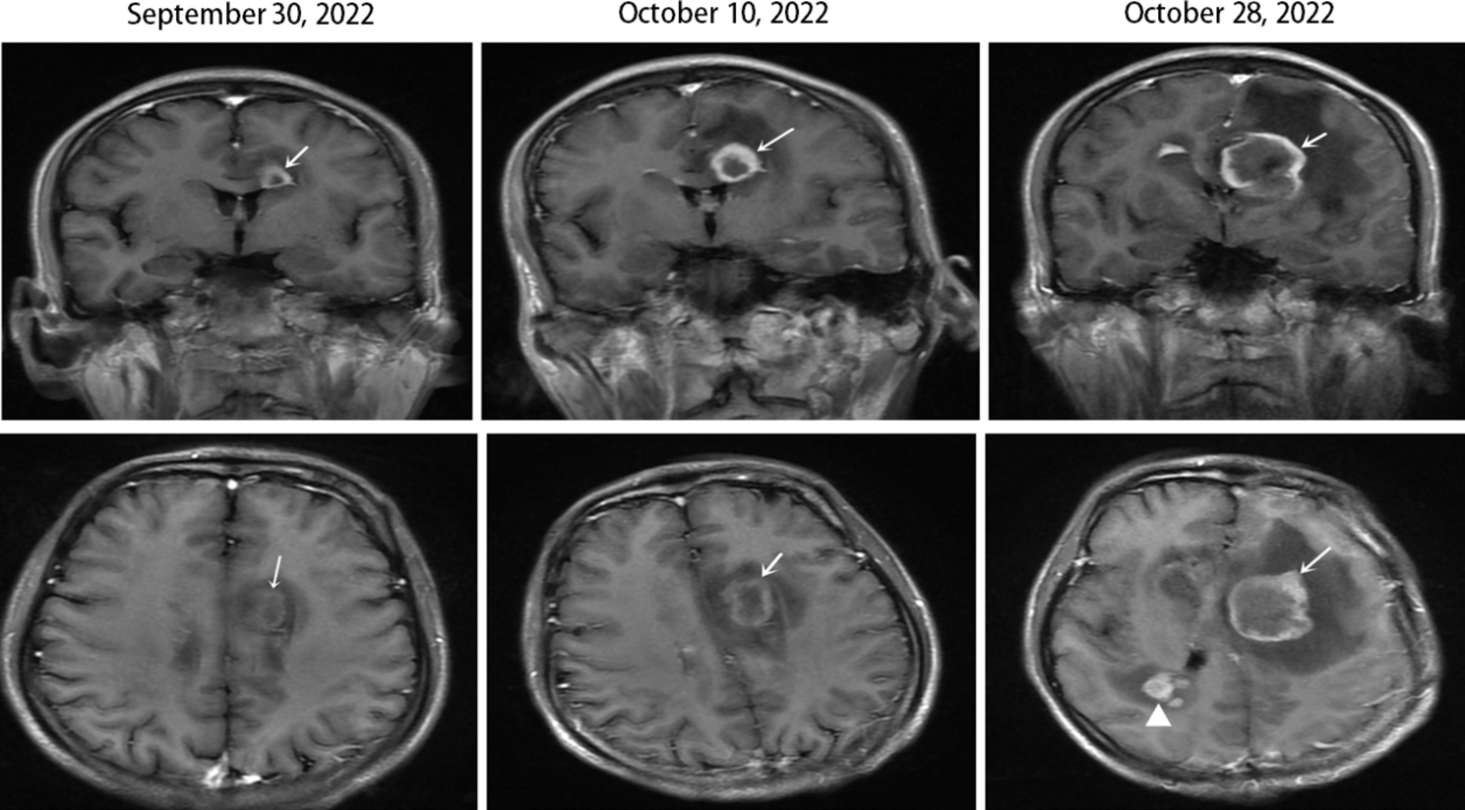
MRI of the brain of our case. The upper row are coronal-enhanced images and the lower row are axial-enhanced images. We can see the ring strengthening lesion in the left lateral ventricle para frontal angle involving the callosum (white → is the strengthening lesion) with central necrosis and perilesional edema. Both MRIs on October 10 and October 28 show progressive enlargement of the lesion with worsening of perilesional edema, and the white △ arrow shows a new lesion in the right lateral ventricle of the right parietal lobe

On October 28, the patient was conscious, slightly slow to respond, unable to answer verbally, and had urination incontinence. In addition, left upper limb muscle strength was grade III, right upper limb and lower limb muscle strength was grade I, and both toe joints can complete command flexion and extension activities. Brain MRI progressed significantly compared with images on 2022-10-10, and lymphoma could not be excluded (Fig. 2). The Methylprednisolone needle 40 mg intravenously drip once a day to reduce intracranial lesion edema, and the mannitol needle 125 ml intravenous drip q8h to relieve cranial pressure. On November 11, however, the lesion size progressed, and the peripheral edema improved slightly. The Methylprednisolone needle was reduced to 30 mg intravenously dripped once a day. The patient still had a fever of 38.4℃, and we switched fluconazole to voriconazole 0.2 g BID for strengthening antifungals. The patient had an average temperature on November 21. On November 27, methylprednisolone tablets were reduced to 16 mg orally once a day. At 2:50 on December 1, 2022, the patient died due to cerebral herniation. On December 15, the brain tissue culture detected acid-fast bacilli. Targeted DNA detection identifies it as *M. avium* (*M. avium* subsp. avium, *M. avium* subsp. paratuberculosis, *M. avium* subsp. hominissuis).

## Discussion

**Fig. 3:**
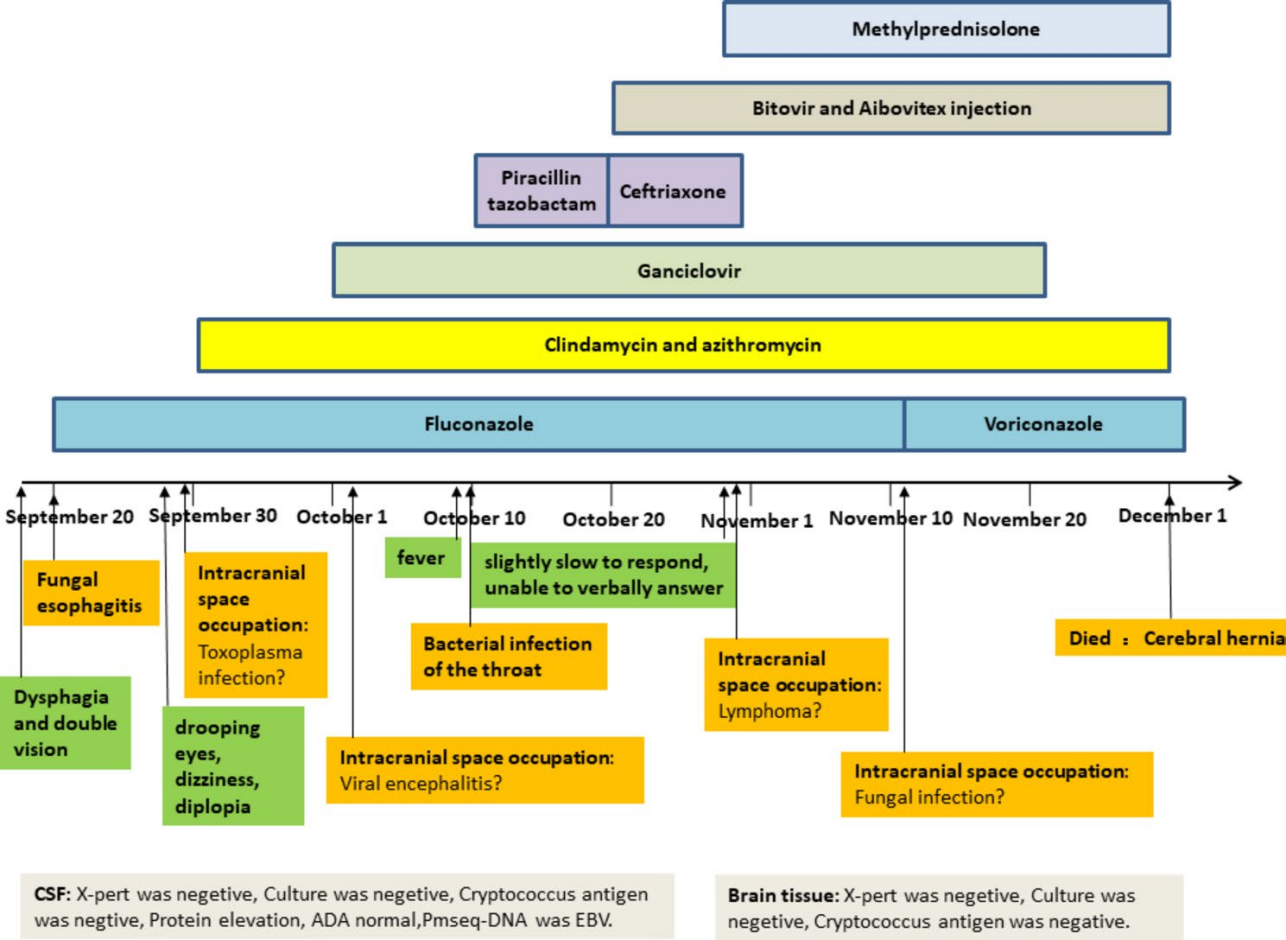
Clinical course, CSF and brain tissue findings, and therapy for our case

There was no evidence of DMAC or any typical symptoms in this case. We empirically diagnosed and treated toxoplasmosis encephalopathy, fungi, and lymphoma. However, the intracranial lesions continued to grow larger and more progressive. Due to the rapid progression of the disease, the patient died (Fig. 3). Finally, we obtained the etiological basis of MAC. The timely diagnosis of MAC involving CNS is difficult, especially for localized cerebral disease, due to difficulty obtaining intracranial tissue and longer culture times.

The most common presentations are DMAC and localized lymphadenitis. Other forms including skin, soft tissue, bone, liver, and spleen disease is rare in HIV patients [5]. DMAC has an incidence of two events per 1000 patient-years and a high mortality rate if not correctly diagnosed and treated in patients living with HIV (PLHIV) [6]. However, the study of MAC involving CNS is minimal. A large-scale study was conducted to identify cases of CNS infections due to NTM in 2011 and only found 15 out of 5960 (< 1%) patients with NTM CNS infection. Among the 15 patients, 4 cases were MAC infection HIV positive patients [7]. We made an extensive search in the English literature only ten additional cases with intracranial infection caused by MAC in HIV-infected patients (Table 1) [4, 8–16]: 9/10 patients were male, and the median age was 38 years (range 24–59 years). The median CD4 count was only 47 cells/µl (range 2-210 cells/µl). MAC was isolated from brain tissue in nine of the ten patients, and only one from CSF presented meningitis. 6/9 patients had a history of DMAC, and 8/10 had initiated ART. Among them, six patients had been diagnosed with MAC-related IRIS. MAC-related IRIS includes paradoxical worsening of treated opportunistic infections or unmasking of previously subclinical untreated infections during the initial months or even years of ART [17]. Five patients were paradoxical MAC infections, and we think one case described by Verma et al. [9]. may present unmasking IRIS. Berger et al. [11]. thought the brain abscess in their case had occurred by chance at the time of HAART initiation and may not be related to IRIS. We can find that localized cerebral disease always happens after a previous history of disseminated MAC infection accompanied by IRIS. Receiving ART after the interruption of anti-MAC therapy, relapsing MAC disease in HIV-infected patients is very low [18], and relapses are often observed in HAART failure and MAC-IRIS [8]. MAC-related IRIS is primarily benign and self-limiting, but severe cases and death had been described [19]. Three patients received steroids for possible paradoxical MAC IRIS after ART initiation [8, 14, 16], and the optimal duration of steroids for CNS MAC IRIS is still unknown. There were no well-defined terms for MAC brain abscess treatment. The 3-drug regimen of a macrolide (azithromycin or clarithromycin), ethambutol, and rifabutin is a primary treatment option for DMAC [20]. The cases in Table 1 provide details of the treatment course, and the antibiotics were provided for 6 to 24 months until the clinic and radiography improved.

**Table 1:** Summary of cerebral Mycobacterium avium complex infection in HIV-infected patients

Case (Reference)	Age/Sex	Year	CD4 cells/µl	Site	Presentation	Diagnostic methods	Duration of HAART	History of DMAC	IRIS	Treatment (Duration)	Outcome
1 [14]	49/M	2021	63	Parietal	abscess	Brain tissue universal broad-range PCRs	Inconsistent	No	Yes	AZM, RFB, EMB, MFX, steroids (> 22 mo)	Alive at 23 mo, MRI unchanged after 15 mo
2 [16]	45/M	2020	108	Parietal, temporal	abscess	Brain tissue universal broad-range PCRs	Inconsistent	Yes	Yes	RIF, INH, PZA, EMB, CLR, [→AZM], steroid taper (NR)	Alive at 6 mo
3 [15]	59/M	2017	20	Temporal	abscess	Brain tissue with AFB stain	Inconsistent	NA	NA	RFB, EMB, ERY (6mo)	NA
4 [13]	47/M	2015	22	Thalamus, frontal, temporal, cerebellar	abscess	Brain tissue culture	No	No	No	CLR, CIP [→RIF], EMB (NR)	Alive after 6 mo
5 [8]	24/M	2013	70	Bilateral cerebral and cerebellar hemispheres, deep gray matter, brain stem	meningoencephalitis	CSF culture	17 months	Yes	Yes	LFX, CLR, EMB, RFB, steroids (NR)	Alive and stable on therapy 1 mo later
6 [4]	42/F	2012	14	right frontoparietal region	abscess	Brain pus culture	No	No	No	CLR, EMB (NR)	Improved CT brain imaging after 1 week; no follow-up reported
7 [9]	33/M	2009	2	Frontal, parietal, occipital	abscess	Brain tissue with AFB stain and culture	6 weeks	Yes	Yes	EMB, RIF, AMK (NR)	Deceased; transitioned to palliative care
8 [10]	36/M	2005	170	right temporal lobe, left temporoparietal	abscess	Brain tissue culture	2 years	Yes	Yes	AZM, EMB, RFB (> 21 mo)	Resolution of lesions after 10 mo
9 [11]	40/M	2004	31	left occipital lobe	abscess	Brain tissue culture and DNA Accuprobe	2 months	Yes	No	CLR, EMB, CIP (6mo) → AZM, EMB (NR)	Alive at 10 mo, with resolution of lesion on CT
10 [12]	35/M	2001	210	left frontal lobe	abscess	Brain tissue culture, AFB stain and PCR	2 years	Yes	Yes	RFB, INH, PZA, EMB, CLR [6wk] → RFB, INH [NR]	Alive after 24 mo
Our case	31/M	2022	5	left lateral ventricle parafrontal angle	abscess	Brain tissue culture and Targeted DNA	No	No	No	Steroids (1 mo)	Died

AFB: acid-fast bacilli; DMAC: disseminated MAC; HAART: Highly active antiretroviral therapy; IRIS: immune reconstitution inflammatory syndrome; AMK, amikacin; AZM, azithromycin; CFZ, clofazimine; CLR, clarithromycin; CSF, cerebrospinal fluid; EMB, ethambutol; ERY, erythromycin; INH, isoniazid; KAN, kanamycin; LFX, levofloxacin; MAC, Mycobacterium avium complex; MOX, moxifloxacin; NR, not reported; PZA, pyrazinamide; RFB, rifabutin; RIF, rifampicin NA: not available

However, there was no history of disseminated MAC infection, and our patient’s localized cerebral disease was detected before ART initiation. The present case is infrequent and timely diagnosis is difficult. MAC is a nonmotile, non-spore-forming, gram-positive acid-fast bacillus and slowly growing mycobacteria; it takes about 10 to 20 days to develop mature colonies [21]. In our case, repeated blood cultures were negative, and the positive results of brain tissue culture took a long time. Several cases of AIDS patients with brain lesions due to M. avium infection had reported negative cultures of blood [13, 19], which may be caused by the low level of the bacterial load. We speculated the reason for the low level of the bacterial load, which may be impacted by immune function in HIV patients. Furthermore, the spectrum of CNS is vast in PLHIV. Intracranial infection is reported to be caused by pathogens like *Toxoplasma gondii, Aspergillus spp, Nocardia spp, Mycobacterium spp, and Cryptococcus neoformans*. In addition, lymphoma is also common. Therefore, intracranial infection involving MAC is not common and easy to ignore. We used the PMseq test of high-throughput gene detection in brain tissue and did not get effective results. After the positive culture result of brain tissue, we tried the targeted DNA detection in brain tissue, hsp65 was used to amplify and sequence mycobacterial DNA, which confirmed MAC in our case, but it was too late.

## Conclusion

MAC infection is challenging to diagnose in immunocompromised patients, particularly intracranial infections. The clinician should obtain as many tissue samples as possible for atypical symptoms and no etiological basis. If routine culture cannot obtain positive results, molecular biology methods are necessary to clarify the diagnosis as soon as possible so that patients can obtain effective treatment.

## Data Availability

No datasets were generated or analysed during the current study.

## Abbreviations

MAC: Mycobacterium avium complex
CNS: Central nervous system
IRD: Immune reconstitution disease
DMAC: Disseminated MAC
IRIS: Immune reconstitution inflammatory syndrome
NTM: Nontuberculous Mycobacterium
AIDS: Acquired immunodeficiency syndrome
cART: Combined antiretroviral therapy
CSF: Cerebrospinal fluid
AFB: Acid-fast bacilli
